# Chitosan-Loaded Vanillin Nanoformulation as an Edible Coating for Post-Harvest Preservation of Indian Gooseberry (Amla)

**DOI:** 10.3390/foods15020395

**Published:** 2026-01-22

**Authors:** Monisha Soni, Archana Kumari, Aarohi Singh, Sangeeta Kumari, Umakant Banjare, Nawal Kishore Dubey, Abhishek Kumar Dwivedy

**Affiliations:** Laboratory of Herbal Pesticides, Centre of Advanced Study (CAS) in Botany, Banaras Hindu University, Varanasi 221005, India; monishasoni1@bhu.ac.in (M.S.);

**Keywords:** edible coating, Indian gooseberry (amla), vanillin, chitosan, post-harvest loss

## Abstract

This is the first investigation that attempts to synthesize chitosan-loaded vanillin nanoformulation (vanillin-Nf) as a novel edible coating agent to prolong the storage life of Indian gooseberry (amla). Different concentrations of vanillin were encapsulated into chitosan via ionic gelation approach using sodium tripolyphosphate as a cross-linker. Vanillin-Nf 1:1 (*w*/*v*) exhibited maximum loading capacity (2.502 ± 0.008%) and encapsulation efficiency (54.483 ± 1.165%). The physico-chemical characterization of vanillin-Nf through SEM, DLS, FT-IR, and XRD techniques confirmed effective incorporation of vanillin into the chitosan biomatrix and formation of spherical nanocapsules, with a mean particle size of 232.83 nm, zeta potential +69.66 mV, and polydispersity index 0.296. The in vitro release profile of vanillin exhibited a biphasic and regulated release pattern. The application of vanillin-Nf as an edible coating solution on amla (*Phyllanthus emblica* L.) fruits was highly effective in reducing decay incidence up to 42.84% and extended their shelf-life to 15 days at 25 ± 2 °C. The vanillin-Nf coating significantly reduced weight loss in amla fruits (24.39 ± 1.02%) in comparison to control. In addition, vanillin-Nf coating also helped in preserving the key quality parameters, including pH, chlorophyll content, total soluble solids, total phenols, and antioxidant capacity of Indian gooseberries to a substantial extent at the end of storage. Collectively, our findings indicate that vanillin-Nf coating is an effective post-harvest approach for controlling decay, prolonging shelf-life, and maintaining the nutritional attributes of Indian gooseberries, highlighting its potential for commercial application in the food and agriculture industry.

## 1. Introduction

*Phyllanthus emblica* L., popularly known as Indian gooseberry or aonla or amla, is a fruit from the family Euphorbiaceae. The fruit is round or globular with a greenish-yellow appearance, known for its distinct sourness and astringent flavour [1]. It is native to India, Southeast Asia, Iran, China, and Pakistan [2]. It is among the most commercially significant fruit crops in India, recognized for its nutritional, medicinal, and nutraceutical properties. The fruit is enriched with vitamin C and was widely used during the COVID-19 pandemic for boosting immunity. It is also an important ingredient in traditional hair and skin care formulations and in the culinary, cosmetic, and pharmaceutical industries [3,4]. Due to high astringency and acidity, consumers do not eat the fruits in their raw form. This creates a huge scope for processing amla and making a variety of products such as Chyawanprash, murabbas, nutritional bars, pickles, candy, jam and jelly, powder drink mixes, yoghurts, and dietary supplements [5]. Amla has a huge demand in both domestic and international markets. India is the leading producer and the biggest exporter of amla and amla extracts to countries like the US, Japan, Nepal, Bangladesh, Malaysia, Germany, and Netherlands. However, the export potential of amla is constrained by its high perishability, inadequate infrastructure and storage facilities, non-uniform grading and packaging, and stringent quality and phytosanitary standards imposed by the importing countries [3,6]. The mature amla fruits have a short life span of 5–6 days accounting for 30% to 40% of post-harvest losses. The fruit is vulnerable to several fungal diseases such as anthracnose, fruit rot, and soft rot. The major fungal pathogens responsible for post-harvest deterioration of amla include *Colletotrichum gloeosporioides*, *Penicillium digitatum*, *P. islandicum* (blue mould), *Phomopsis phyllanthi*, *Aspergillus niger*, *A. flavus*, *A. fumigatus*, *Rhizopus stolonifer*, etc. [7,8]. The infection caused due to blue mould can result in annual production loss up to 50%. Fruit production is negatively impacted by fungal diseases because they reduce fruit quality and marketability. Considering the immense health benefits and economic significance of Indian gooseberries, there is an urgent demand to minimize these post-harvest losses.

Different traditional methods are being employed for post-harvest management of amla which include drying techniques (solar drying, oven drying, freeze drying), hot water treatments (dipping amla at 60 °C for 2 min in hot water prevents contamination from *A. niger*), UV-C exposure, preservation through processing (making amla candies, jams, pickles, etc.), canning, use of perforated plastic packaging, vacuum packaging, and chemical preservatives such as calcium chloride, calcium nitrate, Bavistin, and Bayleton [8]. However, all these management practices have some drawbacks that are either detrimental to human beings or the environment. Since consumers are becoming more selective towards their food choices, the food industry is facing stringent regulations. Hence, there is a need to develop sustainable and environmentally friendly approaches to enhance the shelf-life of amla. Plant products, including essential oils and their components, are maximally explored for preservation purposes as an edible coating. Vanillin (VN), or 4-hydroxy-3-methoxybenzaldehyde, a primary constituent of vanilla, is a natural flavouring agent used in the food, beverage, cosmetic, and pharmaceutical industries [9]. Recently, it has attracted considerable interest for use in food-packaging applications owing to its antioxidant and antimicrobial properties that help keep food fresh for a longer duration by preventing spoilage and quality loss. Moreover, vanillin has been approved by the FDA (Food and Drug Administration) and is classified as GRAS (Generally Recognized As Safe), supporting its suitability for use in food and biomedical applications and highlighting its potential as a reliable option for active food packaging systems [10].

Nanoencapsulation of bioactive components into a suitable biopolymeric matrix and their application as an edible coating for food preservation offers clear advantages over conventional coatings by enhancing solubility and stability of bioactive compounds, protecting against degradation, and enabling their controlled release during storage. The nanoscale size improves dispersion, coating uniformity, enhances antimicrobial efficacy, masks the undesirable taste, and improves the barrier properties against moisture loss and gaseous exchange, thereby delaying senescence and extending the shelf-life [11,12]. Additionally, the traditional coatings may alter the appearance of the coated product, whereas nanoemulsions form a transparent, inconspicuous layer, which allows consumers to visually assess the product quality for acceptance [13]. Among different coating agents, chitosan (CS) is widely recognized as a highly effective, edible, and biocompatible coating material for prolonging the shelf-life and preserving the quality of fruits and vegetables [14]. It is a natural polysaccharide derived from the deacetylation of chitin, composed of repeating units of β-(1,4)-2-amino-D-glucose and β-(1,4)-2-acetamido-D-glucose linked by 1,4-β-glycosidic linkage. It is non-toxic in nature, biocompatible, biodegradable, versatile, cost-effective, and possesses film-forming properties which is ideal for food packaging applications [15,16]. Although, chitosan-based coatings and vanillin have individually been explored for post-harvest applications, their synergistic behaviour in a nanocomposite system, particularly regarding controlled release mechanisms, interaction with fruit physiology, and long-term efficacy during storage, is less explored.

Hence, the objective of the present study was to develop a chitosan-based vanillin nanoformulation (vanillin-Nf) and to investigate its efficacy as an edible coating in preventing fungal decay and enhancing the shelf-life of Indian gooseberries (amla). The vanillin-Nf was physico-chemically characterized through SEM (scanning electron microscopy), DLS (dynamic light scattering), FT-IR spectroscopy, and XRD (X-Ray Diffraction) techniques to confirm successful loading of vanillin into chitosan nanoemulsion. Further, the effect of vanillin-Nf as an edible coating on key quality parameters such as weight loss, total soluble solids, pH, chlorophyll content, total phenols, antioxidant activity, and decay incidence of Indian gooseberries was examined over a 15-day storage period at 25 ± 2 °C to determine its practical applicability as a novel, innovative, and eco-friendly coating solution for reducing post-harvest loss and enhancing the shelf-life of Indian gooseberry.

## 2. Material and Methods

### 2.1. Chemicals, Reagents, and Raw Materials

All chemicals and reagents, such as low-molecular-weight chitosan (deacetylation 90%), molecular weight 10–150 m. Pas, vanillin, Polysorbate-80, Tween-20, acetic acid (99.9% pure), sodium tripolyphosphate anhydrous (Na-TPP), phosphate-buffered saline (PBS), ethyl acetate, DCM (dicholoromethane), sodium carbonate (Na_2_CO_3_), methanol, ethanol, 2,2-diphenyl-1-picrylhydrazyl (DPPH), dimethyl sulfoxide (DMSO), Folin & Ciocalteu’s Phenol Reagent AR, and the culture media used for fungal growth, namely Potato Dextrose Agar (PDA) [Composition: Dextrose (20 g), Agar (15 g), Potato infusion (200 g) dissolved in 1000 mL sterile distilled water] were of analytical grade and procured from Hi-media Laboratories Pvt. Ltd. and SRL (Sisco Research Laboratories Pvt. Ltd.), Maharashtra, India.

A saturated solution of vanillin was prepared by mixing it with DMSO and ethyl acetate (2:3 *v*/*v*). Indian gooseberry (*Phyllanthus emblica* L.) was purchased from the local market of Varanasi (Pahariya). The fruits were collected 48 h post-harvesting and stored at 25 °C before setting up the experiment.

### 2.2. Preparation of Vanillin-Loaded Chitosan Nanoformulation (Vanillin-Nf)

**Figure 1:**
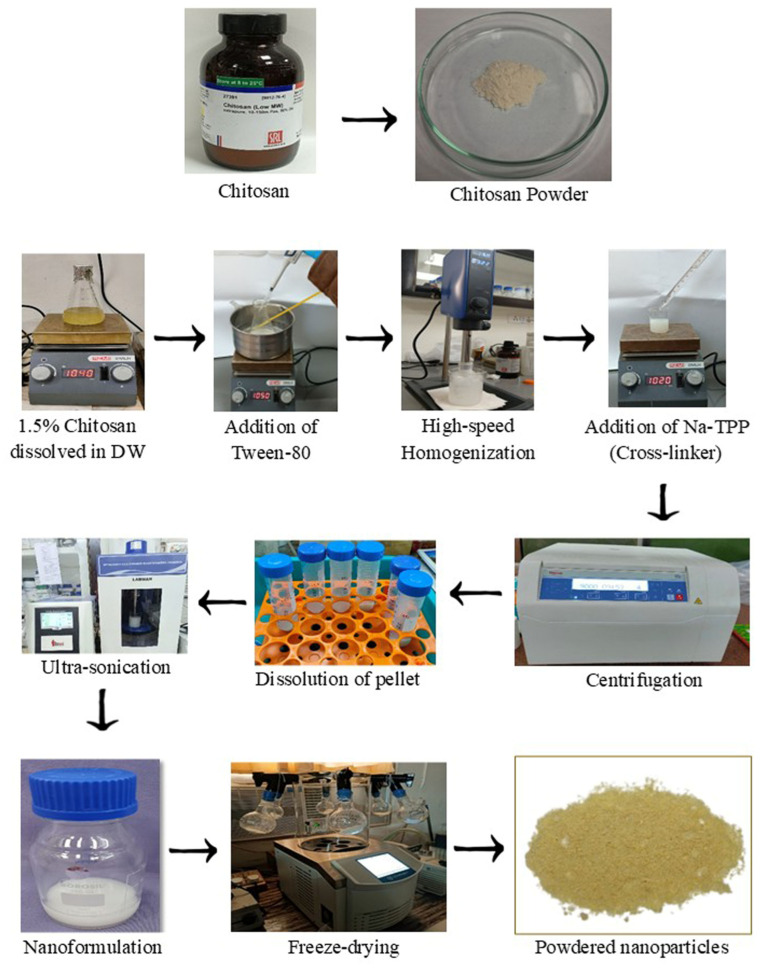
Preparation of vanillin nanoformulation (vanillin-Nf) using chitosan as a nanocarrier.

Modified ionic gelation technique was used to formulate vanillin-Nf (Figure 1) as reported by Yoksan et al. [17]. Chitosan (1.5% *w*/*v*) was dissolved in 1% (*v*/*v*) acetic acid under continuous stirring at 25 °C overnight. Thereafter, Polysorbate 80 was mixed into chitosan solution and agitated in a water bath at 45 °C for 60 min until uniformly homogenized. Different ratios of chitosan and vanillin (*w*/*v*) were formulated (Table 1) by adding a definite volume of vanillin in 4 mL DCM and dispensed into the chitosan solution during the homogenization process occurring at 13,000 rpm for 10 min in an ice bath. The ratio (1:0) served as the chitosan control (CS-CNT), devoid of vanillin. Equal volumes of Na-TPP solution (4 mg/mL) were added dropwise into the homogenized emulsion and stirred for 40 min. The formed nanoparticles were collected through high-speed centrifugation at 10,000 rpm at 4 °C for 15 min. The collected nanoparticles were washed twice with double-distilled water (DDW) to remove unencapsulated vanillin followed by dissolution in distilled water. The nanoformulation was then sonicated using an ultrasonicator (LABMAN PRO650, North Yorkshire, UK) and lyophilized at −53 °C for 60 h. The powdered nanocapsules were used for physico-chemical characterization.

### 2.3. Evaluation of Loading Capacity (LC%) and Encapsulation Efficiency (EE%)

The % LC and EE of vanillin-Nf for different ratios was determined according to the following equations:

$$\%\;LC=\frac{W_{en}}{W_{t}}\times 100$$

$$\%\;EE=\frac{W_{en}}{W_{i}}\times 100$$

where *W_en_* is the amount of vanillin encapsulated into vanillin-Nf, *W_t_* is the total weight of vanillin-Nf, and *W_i_* is the initial weight of vanillin added during nanoemulsion formation. The amount of vanillin encapsulated into vanillin-Nf (*W_en_*) was determined by spectrophotometric method. Vanillin-Nf (0.3 mL) was properly mixed with ethyl acetate (2.7 mL) followed by centrifugation for 12 min at 13,000 rpm at 4 °C. Thereafter, the supernatant was used to record the absorbance at λ_max_ = 273 nm using UV-Vis spectrophotometer (Shimadzu UV-1900i, Kyoto, Japan). Vanillin was dissolved in ethyl acetate to construct the calibration curve (y = 0.0595x − 0.0097; R^2^ = 0.9723). The ratio with maximum % LC and EE was selected for detailed studies.

### 2.4. Characterization of Vanillin-Nf

The freeze-dried nanoformulation (vanillin-Nf) was characterized to determine its morphological characteristics through scanning electron microscopy. For SEM analysis, 0.5 mg of powdered CS-CNT and vanillin-Nf were dispersed in double-distilled water (10 mL) and subjected to sonication (5 min) in an ice bath. A drop of the resulting solution containing chitosan nanoparticles (devoid of and loaded with vanillin, respectively) was placed on a glass slide (1.5 cm × 1.5 cm) and allowed to dry overnight at room temperature. Thereafter, the dried nanoparticles were coated with gold sputter and examined under high-resolution FE-SEM (Carl Zeiss, Oberkochen, Germany, Model: GEMINI 560). The mean particle size, polydispersity index (PDI), and zeta potential of CS-CNT and vanillin-Nf were determined through DLS using a ZetaSizer. For analysis, the samples were diluted with double-distilled water at a 1:10 (*v*/*v*) ratio, and the measurements were carried out at room temperature (25.1 °C). The crystallographic properties of chitosan, CS-CNT, and vanillin-Nf were analyzed using the XRD technique (Rigaku Miniflex 600 (Tokyo, Japan)). All the samples were scanned over a 2θ range of 5° to 50° with a scan rate of 5° min^−1^ and step size of 0.02° min^−1^. FT-IR spectroscopy was performed to elucidate the chemical structure of chitosan, CS-CNT, vanillin, and vanillin-Nf in a wave number ranging from 400 cm^−1^ to 4000 cm^−1^. Before analysis, each sample was thoroughly ground with KBr and compressed to form pellets; 16 scans with a resolution of 4 cm^−1^ were obtained for each spectrum. Each characterization technique (SEM, DLS, XRD, and FTIR) was carried out in triplicate (*n* = 3) to ensure reproducibility and reliability of the results.

### 2.5. Release Kinetics of Vanillin-Nf

The release characteristics of vanillin from vanillin-Nf were studied in a dissolution medium of 40% ethanolic PBS (pH 7.4) following the protocol of Hosseini et al. [18] with minor modifications. Vanillin-Nf (0.3 mL) was dispersed in 2.7 mL ethanolic PBS, thoroughly mixed, and then centrifuged for 10 min at 9000 rpm at 25 °C. Aliquots of 2 mL were taken out for spectrophotometric analysis at 232 nm at specific intervals (0–144 h) and replaced with an equivalent volume of fresh dissolution medium. The cumulative amount of vanillin released in the supernatant was calculated in percent using the given equation:

$$\%\;Cumulative\;release\;of\;vanillin=\sum_{t=0}^{t}\frac{W_{t}}{W_{i}}\times 100$$

where t is the release time, W_t_ denotes the amount of vanillin released at each sampling time, and W_i_ is the initial weight of vanillin loaded into vanillin-Nf.

### 2.6. Isolation and Culture of Fungal Strains

Fungal pathogens were isolated using the method of Chaudhary et al. [19] with slight modifications. The infected amla fruits showing typical symptoms were used for the isolation of fungal pathogens. The rotten fruits were transferred to sterilized tissue culture containers containing PDA (20 mL) and incubated for 48 h at 27 ± 2 °C in a BOD incubator. Later, the pure culture of fungal pathogens was obtained using the streaking method. Identification of isolated fungal pathogens was performed based on their morphological characteristics (colony morphology, colour, pattern of mycelial growth, spore’s characteristics, etc.) using a compound microscope (Labomed vision 2000, Fremont, CA, USA) and by referring to “A Manual of Soil Fungi: Gilman Joseph C.” and an internet database and comparing with those provided in the literature. All the isolated fungal cultures were preserved on PDA slants and stored in a refrigerator at 4 °C for conducting further experiments.

### 2.7. Assessment of Antifungal Efficacy of Vanillin-Nf

The antifungal activity of vanillin-Nf was evaluated against all fungal isolates obtained from mycoflora analysis of amla fruits using the poisoned food assay following the protocol of Geethanjali et al. [20]. In brief, different concentrations of the vanillin-Nf ratio 1:1 (*w*/*v*) were added into sterile PDA media, after which different fungal pathogens were aseptically inoculated using 5 mm discs. The Petri plates without vanillin-Nf were considered as control. Both the treated and control Petri plates were incubated in a BOD incubator at 27 ± 2 °C for 7 days. The minimum inhibitory concentration (MIC) was defined as the lowest concentration of vanillin-Nf at which complete fungal growth inhibition was observed.

### 2.8. Practical Applicability of Vanillin-Nf as an Edible Coating on Indian Gooseberry

Freshly harvested amla fruits were procured from the local market of Varanasi, Uttar Pradesh, and transferred to the laboratory. Later, they were segregated on the basis of uniform shape, size, colour, and if they were free from any bruising or damage. All the fruits were properly washed with distilled water and kept under UV light for 10 min. Thereafter, the amla fruits were divided into three treatment groups containing 20 fruits in each set: the 1st group was coated with double-distilled water (control), the 2nd group was coated with chitosan nanoemulsion devoid of vanillin (CS-CNT), and the 3rd group was coated with vanillin nanoformulation (vanillin-Nf). The coating was applied under aseptic conditions in a laminar air flow chamber by dipping amla fruits into the above-mentioned solutions for 1 min followed by air drying for 15 min. Finally, the fruits were incubated in perforated polyethylene containers at 25 ± 2 °C, and their quality was assessed at the 0, 5th, 10th, and 15th day of storage.

### 2.9. Determination of Change in Weight and pH of Amla

An analytical balance (Shimadzu BX 320 H) was used to track changes in the fruits’ weight during the 15 days of the storage period. The results were expressed as % weight loss calculated using the equation provided below. For determining the pH of amla fruits, 20 g of fruit was homogenized using a blender in 40 mL of distilled water, and the resulting solution was used for pH measurement by Cyberscan PH Tutor Meter.

(%) Weight loss=(Wi−Wf)/Wi×100

where Wi is the initial weight of amla and Wf is the final weight.

### 2.10. Total Soluble Solids (TSS)

The filtrate of homogenized fruit pulp was used to analyze the TSS in amla fruits. The TSS content was measured following the protocol of Tomar and Pradhan [21]. A total of 3–4 drops of the filtrate were placed on the glass prism of a handheld refractometer; the readings were taken, and the results were reported in °Brix.

### 2.11. DPPH Radical Scavenging Activity

The antioxidant capacity of the methanolic extract of amla fruit was analyzed by DPPH assay following Braich et al. [22] with slight modifications. The reaction mixture was prepared by adding different concentrations of methanolic extracts of amla fruit pulp in 3 mL of DPPH solution (0.004% (*w*/*v*)) prepared in methanol and incubated in the dark for 45 min. After incubation, a change in the colour (fading) of DPPH solution was observed which was measured in terms of absorbance at 517 nm via spectrophotometer. The percent inhibition of DPPH radical was measured using the given equation:

$$\%\;Inhibition=\;\frac{A_{control}-A_{reaction\;mixture}}{A_{control}}\times 100$$

where *A* is the absorbance at 517 nm.

### 2.12. Chlorophyll a and b Content

Chlorophyll content was estimated using the DMSO extraction procedure following Parashar [23] with minor modifications. The amla fruit from different treatment sets were chopped into small pieces, and 50 mg of tissue from each sample was added into separate tubes containing 10 mL of DMSO. These tubes were incubated over a water bath for 3 h at 65 °C with gentle shaking over a magnetic stirrer. After the pigments were completely extracted, the clear supernatant was used to record the absorbance with the help of a spectrophotometer, using DMSO as the blank. The absorbance for Chl a and Chl b was recorded at 663 and 645 nm, respectively. The optical density values obtained were then used to calculate the chlorophyll content (mg/g) in the amla extract using the following formula:

$$Chl\;a=(12.3\times {Abs}_{663}-0.86\times {Abs}_{645})\times \frac{V}{1000\times W}$$

$$Chl\;b=(19.3\times {Abs}_{645}-3.6\times {Abs}_{663})\times \frac{V}{1000\times W}$$

where *V* refers to the volume of extract; *W* is the fresh weight of the sample taken; and Abs stands for absorbance at 663 nm and 645 nm.

### 2.13. Total Phenolic Content (TPC)

To evaluate the TPC in amla fruit, the method of Padhi and Dwivedi [24] was followed with some modifications. From each treatment group, 1 g of amla was homogenized in 25 mL of methanol (70%) using a sterile blender. Then, the homogenized sample was heated for 30 min at 60 °C following centrifugation at 9000 rpm for 10 min. Thereafter, the supernatant was carefully collected and kept at 4 °C overnight. Later, the supernatant (60 μL) was diluted with double-distilled water (4.75 mL), followed by the subsequent addition of 300 μL of Folin–Ciocalteu reagent and stirring for 2 min. Finally, 20% Na_2_CO_3_ solution (900 μL) was aliquoted to the above solution, vortexed, and allowed to incubate at 25 °C for 60 min. The TPC in amla was quantified based on absorbance readings at 765 nm and reported as mg gallic acid equivalents per 100 g of fresh weight (mg GAE/100 g FW).

### 2.14. Decay Incidence

The procedure of Rai et al. [25] was followed to calculate decay incidence in amla fruit. The decay percentage in CS-CNT, vanillin-Nf-coated, and uncoated (control) amla fruits was calculated by dividing the number of decayed fruits in each treatment set by the total number of fruits at the start of the experiment and then multiplying by 100.

### 2.15. Statistical Analysis

Each experiment was performed in triplicate, with results expressed as mean ± standard error (S.E.). Data processing and analysis were performed using Microsoft Excel 2016 and IBM SPSS Modeller 16.0. Firstly, the data were examined for normal distribution and homogeneity of variances and were found to be suitable for parametric analysis. Thereafter, statistical analysis was performed using one-way ANOVA with Tukey’s post hoc test to assess significant differences among the datasets at a significance level of *p* < 0.05.

## 3. Results and Discussion

### 3.1. Synthesis of Vanillin-Loaded Chitosan Nanoformulation (Vanillin-Nf)

In our study, chitosan was selected as the encapsulating polymer for vanillin due to its film-forming and gelling properties, production of uniform spherical nanocapsules, high encapsulation potential, structural stability, biodegradability, biocompatibility, and regulated release of bioactives in the food system [26,27]. The nanoformulation containing vanillin (vanillin-Nf) was prepared via ionotropic gelation, which involves electrostatic interactions between the protonated amino groups (NH_3_^+^) of chitosan and polyphosphate groups (PO_4_^3−^) of Na-TPP under acidic conditions [28,29]. In this formulation, Na-TPP acted as a cross-linking agent, operated under gentle temperature and pH conditions, thereby minimizing any potential degradation of bioactive compounds and promoting a controlled-release mechanism [30]. To achieve optimal encapsulation efficiency, various chitosan–vanillin ratios (*w*/*v*) (1:0, 1:0.2, 1:0.4, 1:0.6, 1:0.8, 1:1) were tested to determine the most suitable nanoformulation with the maximum fraction of vanillin loaded within the chitosan biomatrix.

### 3.2. % LC and % EE of Vanillin-Nf

Loading capacity is the measure of the amount of vanillin encapsulated per unit weight of chitosan nanoparticle, whereas encapsulation efficiency refers to the fractional amount of functional component (vanillin) trapped inside the chitosan biopolymer [31]. Both % LC and % EE are the key indicators that suggest vanillin has been encapsulated within the chitosan nanoformulation. Table 2 represents the increment in both % LC and % EE on increasing the concentration of vanillin into the chitosan nanoformulation. The maximum % LC and % EE were observed at the chitosan–vanillin ratio (*w*/*v*) of 1:1 viz. 2.502 ± 0.008%. and 54.483 ± 1.165%, respectively. The increasing trend in both % LC and % EE with higher concentrations of vanillin highlights the effectiveness of chitosan-STPP systems in forming stable nanoemulsions. The peak EE % at a 1:1 ratio indicates an optimal balance between the chitosan biopolymer and the functional bioactive component (vanillin), ensuring maximal entrapment, whereas high % LC implies efficient incorporation of vanillin without excessive polymer use, enhancing its delivery potential. A similar trend of increasing % LC and EE was reported by Das et al. [32] during encapsulation of *Cinnamomum camphora* EO into the chitosan biomatrix. The nanoformulation containing the chitosan–vanillin ratio 1:1 (*w*/*v*) demonstrated superior encapsulation and loading efficiencies; therefore, this ratio was selected for further physiochemical characterization, antifungal assay, and analyzing its practical applicability.

### 3.3. Characterization of Vanillin-Nf

#### 3.3.1. Scanning Electron Microscopy

**Figure 2:**
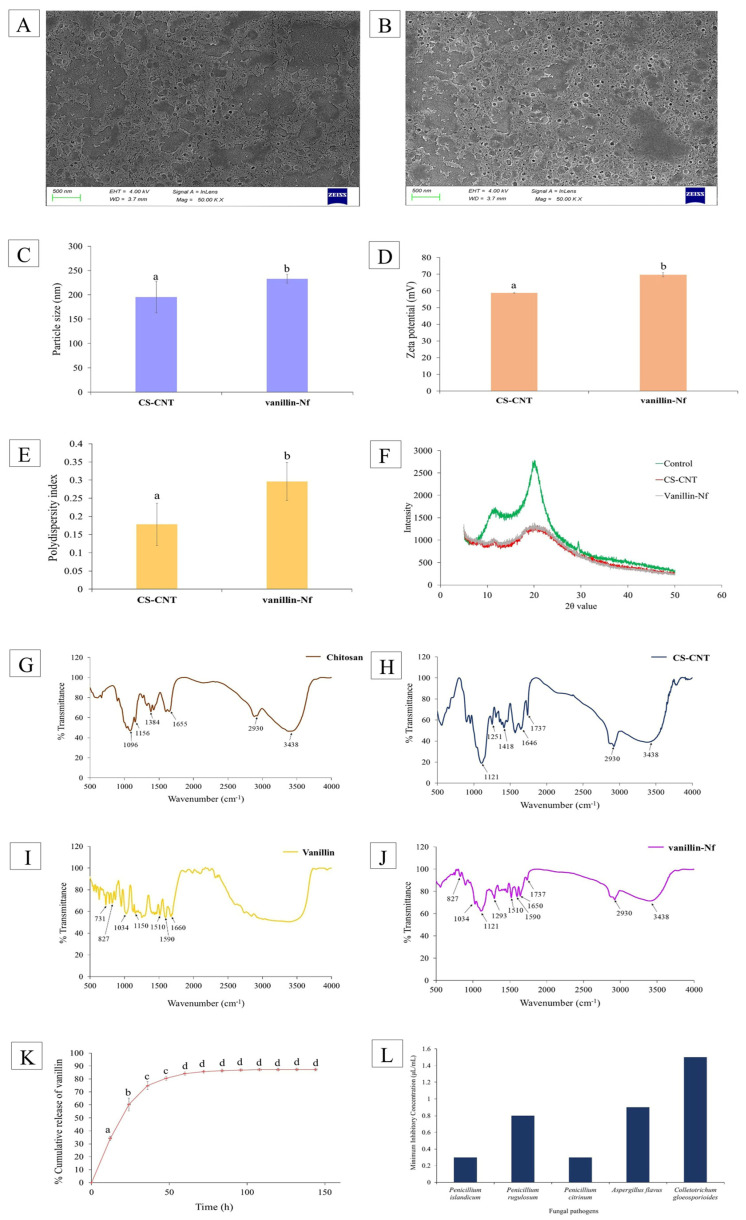
SEM images of (**A**) CS-CNT; (**B**) vanillin-Nf; (**C**) mean particle size (nm) of CS-CNT and vanillin-Nf; (**D**) Zeta potential (mV) of CS-CNT and vanillin-Nf; (**E**) polydispersity index of CS-CNT and vanillin-Nf; (**F**) XRD patterns of chitosan, CS-CNT, and vanillin-Nf. FTIR spectra of (**G**) chitosan, (**H**) CS-CNT, (**I**) vanillin, and (**J**) vanillin-Nf; (**K**) in vitro release behaviour of vanillin from vanillin-Nf; (**L**) antifungal efficacy of vanillin-Nf against isolated fungal cultures [values are mean (*n* = 3) ± S.E.; different letters denote significant differences at *p* < 0.05, based on ANOVA and Tukey’s post hoc tests].

The morphology and surface characteristics of unloaded and vanillin-loaded chitosan nanoparticles were examined using SEM analysis. Figure 2A,B represents the SEM micrographs of CS-CNT and vanillin-Nf, respectively. Both the CS-CNT and vanillin-Nf exhibited well-defined, spherical shapes with good dispersion and structural stability. The analysis of particle size of CS-CNT and vanillin-Nf indicated that vanillin-loaded chitosan nanoparticles exhibited larger diameters as compared to CS-CNT, likely due to successful entrapment and effective loading of vanillin into the chitosan biopolymer. Our findings are in agreement with Cai et al. [33], who observed a larger particle size of chitosan nanoparticles loaded with *Ocimum basilicum* EO as compared to unloaded nanoparticles. Another study by Jiang et al. [34] revealed that the incorporation of *Eucommia ulmoides* seed EO resulted in chitosan nanoparticles with larger dimensions and a more compact morphology than empty chitosan nanoparticles, indicating successful loading of EO into chitosan nanoparticles.

#### 3.3.2. Dynamic Light Scattering

The mean particle size, zeta potential, and PDI of CS-CNT and vanillin-Nf was further confirmed by DLS analysis as presented in Figure 2C–E, respectively. The CS-CNT had a mean particle size of 195.60 nm, while the mean particle size increased to 232.83 nm after encapsulation of vanillin into the chitosan nanoformulation (vanillin-Nf), which is in accordance with results reported by Hasheminejad et al. [35]. The increase in mean particle size of vanillin-Nf is attributed to the swelling of chitosan nanoparticles following vanillin incorporation, which promotes polymer chain relaxation and expansion due to increased electrostatic interactions between vanillin and the chitosan biomatrix [36,37]. Hadidi et al. [38] also reported that there was an increment in the mean particle diameter from 223.2 nm to 444.5 nm after encapsulation of clove EO into chitosan nanoparticles.

Zeta potential and PDI indirectly provide information on colloidal stability. Zeta potential is an important parameter that indicates the degree of electrostatic repulsion between the neighbouring particles within a dispersion system, while the polydispersity index is commonly used to evaluate the distribution of particle size in colloidal suspensions. The low PDI values (near 0) signify a narrower particle size distribution, which is indicative of greater homogeneity and uniformity of particle diameters [39,40]. The particles exhibiting high absolute zeta potential tend to repel each other, preventing particle aggregation and maintaining a narrow particle size distribution which is often reflected by a low PDI. As a result, such systems are likely to be associated with enhanced dispersion stability over time [41]. Conversely, low zeta potential leads to weak repulsive forces that promotes particle aggregation and results in broader size distribution and higher PDI. Hence, zeta potential plays an important role in determining PDI by controlling inter-particle interactions [42]. The nanoparticles with an absolute zeta potential greater than ±30 mV are generally considered stable because of sufficient electrostatic repulsion between particles [43].

The zeta potential values for CS-CNT and vanillin-Nf were recorded as +58.73 mV and +69.66 mV, respectively (Figure 2D), indicating a high positive surface charge and excellent colloidal stability. The increase in zeta potential after encapsulation of vanillin confirms successful incorporation and stronger electrostatic repulsion between nanoparticles, which minimizes aggregation, enhances the colloidal stability of vanillin-Nf, and indicates uniform coating deposition on the fruit surface [44]. Souza et al. [45] reported that the zeta potential for lecithin/chitosan nanoparticles loaded with quercetin was found to be 56.46 ± 1.94 mV, suggesting formation of a stable suspension. The PDI for CS-CNT and vanillin-Nf was found to be 0.178 and 0.296, respectively (Figure 2E), suggesting the formation of a predominantly monodisperse system. These low PDI values further indicate good dispersion stability and a uniform particle distribution within the chitosan nanoformulation.

#### 3.3.3. X-Ray Diffraction

The XRD analysis of chitosan, CS-CNT, and vanillin-Nf is depicted in Figure 2F. The XRD analysis of pure chitosan displayed two prominent peaks at 2θ values of 11.9° and 20.3°, reflecting its semi-crystalline nature. These characteristic peaks are in agreement with earlier findings reported by Azadi et al. [46] and are associated with the ordered packaging of chitosan chains arising from strong intra- and intermolecular hydrogen bonding [47]. In contrast, CS-CNT and vanillin-Nf samples showed a noticeable reduction in peak intensity along with peak broadening, indicating a disruption of the native crystalline structure of chitosan. This structural modification suggests a decrease in crystallinity and a corresponding increase in the amorphous character of the chitosan biomatrix, likely due to intermolecular interactions such as hydrogen bonding, van der Waals forces, and electrostatic and ionic interactions between chitosan, vanillin, and Na-TPP. These intermolecular interactions restrict the molecular mobility of vanillin within the chitosan biomatrix and provide stability to the vanillin nanoformulation [48,49]. Mirsharifi et al. [50] prepared a composite film using almond gum, polyvinyl alcohol, and chitosan incorporated with thyme EO nanoemulsion and observed its XRD patterns. The composite film exhibited an amorphous structure, suggesting effective encapsulation and homogenous dispersion of thyme EO within the polymeric matrix, leading to loss of its native crystalline structure. Another study by da Silva et al. [51] also revealed noticeable modifications in the XRD patterns after the transformation of chitosan and polyvinyl alcohol (PVA) into films incorporated with sweet fennel oil-loaded nanoemulsion. The reduction in crystallinity was observed in all the films, which was attributed to the addition of sorbitol as a plasticizer.

#### 3.3.4. Fourier Transform Infrared Spectroscopy

Figure 2G–J represent infrared diffraction patterns of chitosan, CS-CNT, vanillin, and vanillin-Nf, respectively. The FTIR spectrum of chitosan revealed several absorption peaks that reflect its polysaccharide nature. The characteristic peak around 897 cm^−1^ corresponds to anomeric C-H vibrations, confirming the presence of glycosidic linkages in chitosan biopolymer. The peak at 1096 cm^−1^ and 1156 cm^−1^ is associated with asymmetric stretching of C-O-C bonds [52]. The peak at 1384 cm^−1^ is related to symmetric C-H bending vibrations, 1643 cm^−1^ corresponds to C=O stretching vibration of amide I, and absorption bands at 2877 cm^−1^ and 2930 cm^−1^ denote C-H stretching vibrations of methylene groups. Furthermore, a broad absorption band centred at 3453 cm^−1^ is attributed to the stretching vibrations of O-H and C-H bonds [53]. The formation of CS-CNT was evidenced by a noticeable shift in characteristic spectral peaks along with the emergence of new absorption bands. The shift in characteristic peaks from 1096 cm^−1^ to 1121 cm^−1^, 1156 cm^−1^ to 1250 cm^−1^, and 1655 cm^−1^ to 1646 cm^−1^ suggests electrostatic crosslinking between ammonium groups of chitosan and the P=O group of Na-TPP [54,55]. The FTIR spectra of pure vanillin displayed distinct spectral peaks at 1510 cm^−1^, 1590 cm^−1^, and 1660 cm^−1^, which are attributed to benzene ring stretching vibrations and C=O stretching of the aldehyde functional group [56]. It has been observed that after encapsulating vanillin into chitosan biopolymer, the absorption spectra of vanillin-Nf showed common spectral peaks of CS-CNT and vanillin along with the stretching of some spectral peaks, indicating successful entrapment of vanillin in the chitosan biopolymer. Similar results were reported by Jamil et al. [57], who suggested that all the characteristic peaks of chitosan nanoparticles and cardamom EO appeared in the absorption spectra of cardamom EO-loaded chitosan nanoparticles, indicating efficient encapsulation of cardamom EO.

### 3.4. Release Kinetics of Vanillin from Vanillin-Nf

Figure 2K shows the release curve of vanillin from vanillin-Nf in phosphate buffer (pH = 7.4), which was determined via spectrophotometric method by recording absorbance at 232 nm. A two-step biphasic release pattern was observed, comprising initial burst release and subsequently a gradual/slow-release behaviour as depicted in Figure 2K. The vanillin molecules adsorbed on the surface or located close to the surface of nanoparticles were responsible for causing the first burst release, which was found to be 34.21% and 60.29% at 12 h and 24 h, respectively. Up to 72 h, a rapid release of vanillin was observed (85.69%); thereafter, the release pattern showed a slow release of vanillin from the vanillin nanoformulation. The slow-release pattern might be attributed to the gradual movement of encapsulated vanillin from the nanoparticle core into the PBS solution via pores and channels within the chitosan biomatrix [58]. The enhanced amorphous nature of the chitosan nanoformulation after the loading of vanillin played a crucial role in the sustained release of vanillin, as the disordered arrangement of polymer chains promotes swelling and creates a diffusion-controlled barrier that retards rapid migration of vanillin into the surrounding medium [59]. These observations are in line with the findings of Negi and Kesari [60] who reported that the release behaviour of *Carum copticum* EO from chitosan polymer followed a biphasic process including an initial burst observed for 5 h and then a slow declining phase recorded until 24 h. They suggested that the initial burst was due to the loosely bound or superficially entrapped EO molecules. After 24 h, a steady state was achieved until 72 h followed by a significant decrease as the plateau stage was achieved.

### 3.5. Isolated Fungal Pathogens and Antifungal Activity of Vanillin-Nf

Five species of fungi were isolated during the mycoflora analysis of amla, namely *Penicillium islandicum*, *Penicillium rugulosum*, *Penicillium citrinum*, *Aspergillus flavus*, and *Colletotrichum gloeosporioides*, which were identified through morphological characteristics using a compound microscope. The MIC of vanillin-Nf against *P. islandicum*, *P. rugulosum*, *P. citrinum*, *A. flavus*, and *C. gloeosporioides* was recorded as 0.3, 0.8, 0.3, 0.9, and 1.5 μL/mL, respectively (Figure 2L). The vanillin-Nf exhibited strong antifungal activity against all tested fungal isolates. However, it was found to be most effective against *Penicillium* sps as compared to *Aspergillus* and *Colletotrichum* spp. The antifungal property of vanillin-Nf is primarily due to the synergistic effect of the bioactive component (vanillin) and chitosan and the increased surface area of nanosized particles, enhancing cellular penetration. Moreover, the controlled release of vanillin from the chitosan nanoformulation allows the bioactive component to act for longer periods, leading to enhanced stability and bioavailability, thereby inhibiting fungal growth [61]. The studies of Akhter et al. [62] and Hasanin et al. [63] also suggested that EO nanoemulsions were highly effective in controlling fungal pathogens due to their small size and increased surface area, which enhances their interaction and allows faster penetration of bioactive compounds into the fungal cells.

### 3.6. Practical Applicability of Vanillin-Nf as an Edible Coating on Indian Gooseberry

**Figure 3:**
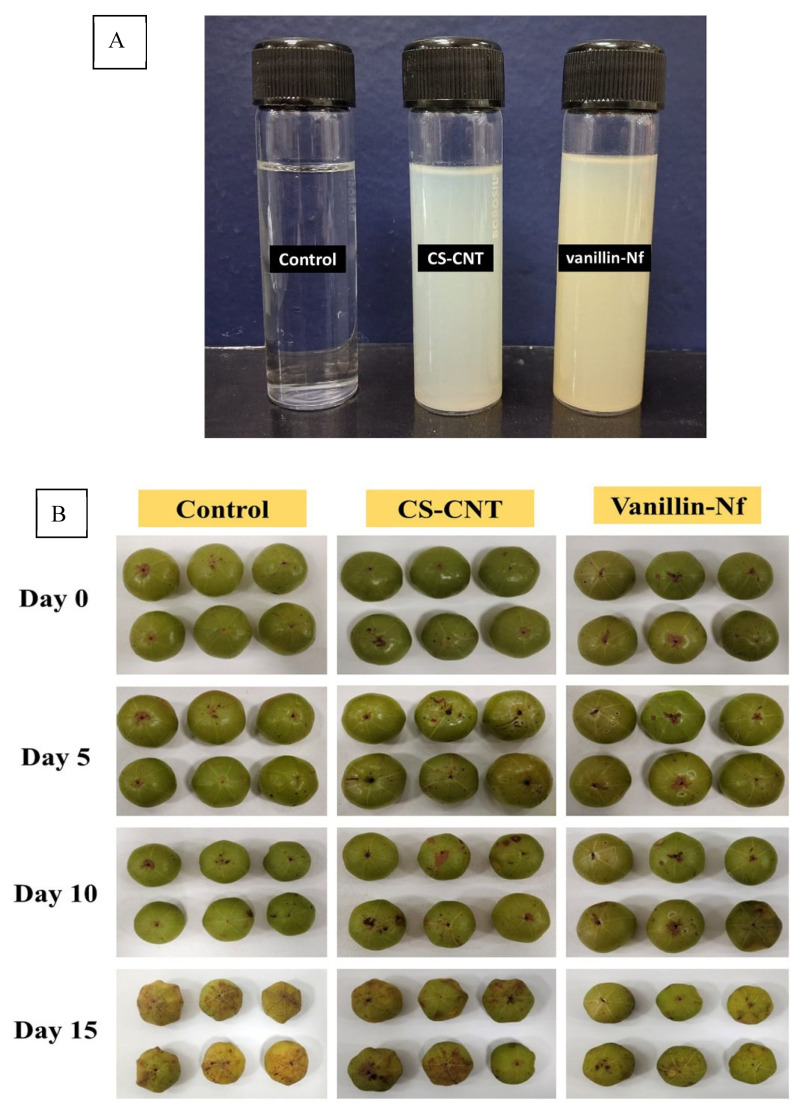
(**A**) Different treatment solutions used to coat amla fruits. (**B**) Effect of vanillin-Nf coating on visual quality and shelf-life of Indian gooseberries stored for 15 days at 25 ± 2 °C.

Indian gooseberry (amla), known for its nutritional and therapeutic value, is highly perishable due to rapid moisture loss, browning, incidence of post-harvest diseases, and degradation of vitamin C during storage. These factors are often associated with surface shrivelling and loss of firmness and nutritional attributes, leading to reduced consumer acceptability and marketability [64]. Hence, in our study, amla fruits were coated with three treatment solutions viz. Control, CS-CNT, and vanillin-Nf as shown in Figure 3A, and their effect on the shelf-life of amla fruits were evaluated during 15 days of storage after harvest (Figure 3B). At day 0, amla fruits in all treatment sets exhibited higher apparent freshness. However, the visual quality deteriorated with storage progression as evident in Figure 3B. After 5 days of storage, noticeable surface shrivelling, loss of firmness, microbial spoilage, and browning were observed in fruits treated with CS-CNT and distilled water (control), which intensified significantly by the 10th and 15th day of storage. In contrast, amla fruits treated with vanillin-Nf retained moisture and intact surface morphology with minimal browning as evidenced on the 15th day of storage in Figure 3B. These findings indicate that vanillin-Nf coating effectively preserved the visual freshness and enhanced the storage life of amla fruits up to 15 days, highlighting its practical applicability as a safe, biodegradable, and edible coating solution for enhancing the shelf-life of amla fruits.

### 3.7. Weight Loss, Total Soluble Solids (TSS), and pH

**Figure 4:**
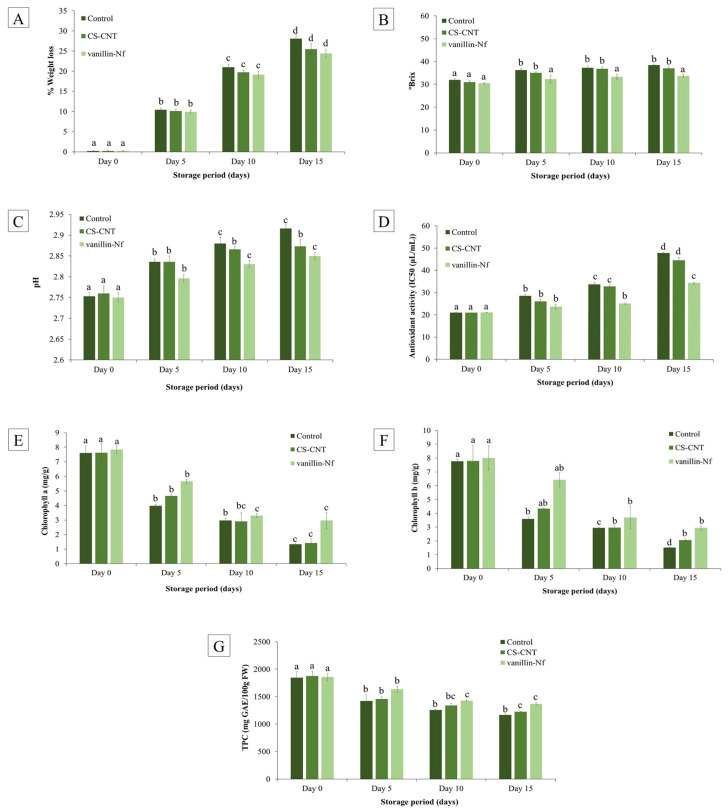
Effect of vanillin-Nf as an edible coating on (**A**) weight loss, (**B**) total soluble solids, (**C**) pH, (**D**) antioxidant activity, (**E**) chlorophyll a content, (**F**) chlorophyll b content, and (**G**) total phenolic content of Indian gooseberries stored for 15 days at 25 ± 2 °C (Different letters denote significant differences at *p* < 0.05, based on ANOVA and Tukey’s post hoc tests).

The most prominent physiological change observed in amla fruits after harvest is moisture loss, which occurs due to accelerated respiration rate and transpiration, leading to wilting and shrivelling. These changes adversely affect the visual quality of amla fruit, leading to a reduction in their market value. These physiological processes are often influenced by factors such as post-harvest treatments and the temperatures at which fruit is stored [65,66]. Figure 4A represents % change in weight of amla fruits coated with different treatment solutions. The results indicate that amla fruits coated with vanillin-Nf exhibited significantly lower weight loss as compared to CS-CNT-coated and control fruits (*p* < 0.05). After 5 days of storage, the control group showed a weight loss of 10.43 ± 0.45%, which further increased to 28.09 ± 0.94% by day 15 of storage. In contrast, all the fruits coated with CS-CNT and vanillin-Nf maintained reduced weight loss throughout the storage period. However, the chitosan coating infused with vanillin was most effective, recording weight loss of 9.91 ± 0.51% on day 5 and 24.39 ± 1.02% by the end of storage. The reduced weight loss in vanillin-Nf-coated amla fruits indicates that the vanillin-Nf coating enhances the barrier properties of fruit, thereby limiting moisture loss and reducing dehydration, which ultimately contributes to improved weight retention. Das et al. [67] suggested that the chitosan nanoemulsion coating containing caraway EO significantly enhanced the quality and safety of bananas during 7 days of storage by effectively minimizing weight loss. Another study by Al-Farsi et al. [68] reported that 1% chitosan coating enriched with grape seed oil was effective in maintaining the moisture levels in date fruits up to 63% in comparison to the control group (51%) over 21 days of storage.

Total soluble solids indicate the sugar content in amla fruits. The TSS often rises during post-harvest storage because the complex polysaccharides are metabolized into simple sugars during respiration along with concentration of fruit juices resulting from moisture loss during transpiration [69], while the shift in pH reflects utilization of organic acids as respiratory substrates. In our study, an increase in TSS was observed with the increase in storage time as depicted in Figure 4B. At day 0, amla fruits in all treatment groups were at similar stages of maturity and age; hence, the TSS values were nearly identical, averaging about 31.1 °Brix. With the progression of storage time, the lowest TSS values were recorded for vanillin-Nf-coated amla fruits, measuring 33.7 °Brix at the 15th day of storage at 25 ± 2 °C. In contrast, the control and CS-CNT-coated amla fruits exhibited higher TSS levels, reaching 38.5 and 37.0 °Brix, respectively, at the end of storage.

Similarly, the pH of amla juice showed a gradual increase with progression of storage time in all treatment groups during 15 days of storage at 25 ± 2 °C (Figure 4C). Statistically significant differences (*p* < 0.05) in pH were observed throughout the storage period, with maximum change recorded in the control samples where the pH of amla juice changed from 2.75 at day 0 to 2.92 at day 15. In comparison, the amla fruits coated with CS-CNT and vanillin-Nf were able to maintain the pH of amla fruits during the end of storage. The pH of amla juice in CS-CNT and vanillin-Nf was recorded as 2.76 and 2.57 at day 0 which rose to 2.87 and 2.85, respectively. The increase in pH of amla fruits in all the treatments may be attributed to the accelerated transpiration and respiration rate because the fruits were stored at 25 ± 2 °C, causing a rapid decline in ascorbic acid content and a consequent rise in pH. The lower weight loss, moderate accumulation of TSS, and relatively stable pH observed in vanillin-Nf-coated amla fruits suggest overall reduction in respiration rate and metabolic activity. This indicates that the vanillin-Nf edible coating acts as a barrier, limiting gaseous exchange and moisture loss, thereby delaying senescence and preserving fruit quality during storage. Sebastian et al. [70] suggested that neem oil-coated amla fruits stored in low-density polyethylene pouches recorded lower TSS values (13 °Brix) in comparison to control samples, reaching 24 °Brix by the 15th day of storage. Tomar and Pradhan [21] also revealed that the amla fruits stored at 25 °C exhibited higher pH values and a significant increase in TSS (*p* < 0.05) than those stored at 6 °C due to accelerated metabolic activity, moisture loss due to transpiration, and ascorbic acid degradation at higher temperatures.

### 3.8. Antioxidant Activity

During post-harvest storage, fruit senescence and ripening are accompanied by accumulation of ROS, which accelerates oxidative stress and leads to deterioration of nutritional quality. Hence, with progression in storage time the antioxidant activity of amla juice declined sharply in all treatments (Figure 4D). The decline in the antioxidant activity in amla fruits during storage can be primarily attributed to progressive degradation of ascorbic acid content over time, as ascorbic acid is the major contributor to the fruit’s overall antioxidant activity [71]. However, the vanillin-Nf coating was able to manage the ROS production in amla fruits and preserved the nutritional content during 15 days of storage. A study by Yang et al. [72] demonstrated that nanoemulsion coating containing eugenol, carvacrol, and cinnamaldehyde effectively maintained higher SOD, CAT, APX, and POD enzyme activities in Nanfeng tangerine during cold storage. Consequently, the nanoemulsion-coated fruits exhibited a greater capacity to scavenge free radicals compared with the control.

### 3.9. Chlorophyll and Total Phenolic Content

The chlorophyll a (Figure 4E) and chlorophyll b (Figure 4F) contents showed a progressive decline during the entire storage period across all treatment groups. However, vanillin-Nf-coated amla fruits were able to maintain the chlorophyll a (2.96 mg/g) and chlorophyll b (2.94 mg/g) content at the 15th day of storage as compared to control and CS-CNT-treated fruits. The reduction in chlorophyll is primarily associated with the activity of chlorophyllase and the pheophytinase enzyme, which play a key role in chlorophyll degradation. A decline in chlorophyll levels in photosynthetic tissues after harvest is an indicator of fruit senescence [73]. Phenols are bioactive secondary metabolites synthesized in plants that help to neutralize reactive oxygen species (ROS) [61]. As a result, fruits with higher phenolic content generally exhibit higher antioxidant capacity. Figure 4G represents change in total phenolic content of amla fruits during 15 days of storage at 25 ± 2 °C in all treatment groups. It was observed that total phenolic content decreased from day 0 (~1860.40 mg GAE/100 g FW) until the end of the storage period in all treatment groups, with a maximum decrease observed in the control group (1165.30 mg GAE/100 g FW) followed by CS-CNT (1226.40 mg GAE/100 g FW), whereas the vanillin-Nf edible coating was able to maintain the level of total phenols (1365.90 mg GAE/100 g FW) in amla fruits at the end of the storage period. The reduction in total phenolic content in uncoated amla fruits may be attributed to an increased respiration rate, enhanced antimicrobial activity, and enzymatic degradation of phenolic compounds during storage [32]. The observed trends in chlorophyll degradation and total phenolic content are closely associated with the management of oxidative stress and progression of senescence during storage. However, a slow decline in chlorophyll content coupled with better retention of phenolic compounds in vanillin-Nf-coated amla fruits suggests the effectiveness of the vanillin-Nf coating in protecting amla fruits against oxidative stress, delaying senescence, and maintaining fruit quality during storage by forming a barrier [74,75]. Our results comply with Kumari et al. [76], who reported a reduction in chlorophyll content in aonla fruits during 15 days of storage. Another study by Dave et al. [77] revealed that edible coating made from soy protein, hydroxymethyl cellulose, and olive oil was able to reduce chlorophyll degradation (*p* < 0.05) in pear fruits stored at 28 ± 5 °C and 60 ± 10% relative humidity as compared to untreated fruits.

### 3.10. Evaluation of Decay Incidence

Decay incidence in amla fruits increased progressively with the advancement of the storage period (Table 3). However, this increase was markedly greater in control fruits than in amla fruits treated with CS-CNT and vanillin-Nf. Among all treatments, the application of vanillin-Nf was most effective in limiting decay in amla fruits, showing superior performance as compared to control and CS-CNT treatments. By day 15, the decay % in the control group was highest (92.42%), followed by CS-CNT-coated fruits (72.61%), while the lowest decay was observed in vanillin-Nf-coated amla fruits (42.84%). These results clearly indicate that the vanillin-Nf coating was the most effective treatment in reducing the decay incidence in amla fruits stored for 15 days at 25 ± 2 °C, indicating efficient antimicrobial potential of vanillin-Nf in preventing post-harvest decay. The edible coating of vanillin-Nf forms a defensive barrier around the amla fruits, which restricts the growth and spread of pathogenic microorganisms. Moreover, the sustained release of vanillin from the chitosan biomatrix leads to enhanced bioavailability of bioactive components for longer periods, thereby reducing decay and enhancing the shelf-life of amla fruits [78]. A significant decrease in decay incidence has been previously reported by Saleem et al. [79] in chitosan–ascorbic acid-coated strawberries. Moreover, Iqbal et al. [80] also observed that a chitosan nanoemulsion-based edible coating containing aloe vera gel and zinc oxide nanoparticles was able to reduce the decay rate in tomatoes (0.85–20.40%) in comparison to untreated tomatoes (1.05–35.20%) stored at 20 °C for a period of 20 days.

## 4. Conclusions

Indian gooseberry (amla), widely known for its medicinal properties, has a limited shelf-life, highlighting the ongoing need to investigate and develop effective strategies to improve its post-harvest shelf-life. In our study, vanillin-loaded chitosan nanoformulation (vanillin-Nf) was developed as an edible coating solution to enhance the shelf-life of amla fruits and to control fungal growth. The vanillin-Nf was effective in inhibiting fungal pathogens associated with post-harvest loss of amla fruits and extended the shelf-life of amla fruits up to 15 days of storage at room temperature (25 ± 2 °C). The vanillin-Nf coating could maintain the physio-chemical properties of amla fruits during storage, including change in weight, pH, total soluble solids, chlorophyll a and b content, total phenols, and antioxidant activity. The coating acted as a protective barrier and also regulated the release of vanillin from vanillin-Nf, which was responsible for its prolonged antifungal activity and reduced decay rate in vanillin-Nf coated amla fruits. Overall, vanillin-Nf represents a sustainable and eco-friendly post-harvest strategy to enhance the shelf-life of amla, though further studies are needed to assess its large-scale commercial applicability and long-term safety.

## Figures and Tables

**Table 1:** Ratios of chitosan: vanillin (*w*/*w*) used for preparing vanillin-Nf.

Ratio of Chitosan: Vanillin (*w*/*v*)	Chitosan (g)	Vanillin (µL)	DCM (mL)	Na-TPP (mg)
1:0	0.3	0.0	4.0	80
1:0.2	0.3	55.90	4.0	80
1:0.4	0.3	111.90	4.0	80
1:0.6	0.3	167.90	4.0	80
1:0.8	0.3	223.80	4.0	80
1:1.0	0.3	279.80	4.0	80

**Table 2:** % loading capacity and % encapsulation efficiency of vanillin-Nf prepared at different ratios of chitosan–vanillin (*w*/*v*).

Chitosan–Vanillin (*w*/*v*)	% Loading Capacity	% Encapsulation Efficiency
1:0	0.000 ± 0.000 ^a^	0.000 ± 0.000 ^a^
1:0.2	0.439 ± 0.051 ^b^	26.220 ± 0.928 ^b^
1:0.4	0.856 ± 0.076 ^c^	29.995 ± 2.849 ^c^
1:0.6	1.050 ± 0.022 ^d^	32.122 ± 1.236 ^d^
1:0.8	1.239 ± 0.081 ^e^	40.996 ± 1.220 ^e^
**1:1**	**2.502 ± 0.048 ^f^**	**54.483 ± 1.165 ^f^**

Note: Data are expressed as mean (*n* = 3) ± S.E. Superscript letters indicate significant differences at *p* < 0.05, as determined by ANOVA and Tukey’s post hoc tests. Values shown in bold correspond to the chitosan–vanillin (*w*/*v*) ratio that resulted in maximum loading capacity and encapsulation efficiency.

**Table 3:** Decay (%) of amla fruits coated with control, CS-CNT, and vanillin-Nf.

Days of Storage	Control	CS-CNT	Vanillin-Nf
0	0.00 ± 0.00 ^a^	0.00 ± 0.00 ^a^	0.00 ± 0.00 ^a^
5	23.87 ± 1.77 ^b^	9.63 ± 0.81 ^b^	4.52 ± 0.54 ^b^
10	58.81 ± 1.78 ^c^	56.41 ± 2.62 ^c^	28.87 ± 1.39 ^c^
15	92.42 ± 1.83 ^d^	72.61 ± 1.18 ^d^	42.84 ± 1.86 ^d^

Note: Data are expressed as mean (*n* = 3) ± S.E. Superscript letters indicate significant differences at *p* < 0.05, as determined by ANOVA and Tukey’s post hoc tests.

## Data Availability

The original contributions presented in this study are included in the article. Further inquiries can be directed to the corresponding authors.

## References

[1] Charoenphun N., Ban Z., Liu L., Paulraj B., Venkatachalam K. (2025). A Review of Post-Harvest Management Practices and Quality Optimization Strategies for Indian Gooseberry (*Phyllanthus emblica* L.). Trends Sci..

[2] Gul M., Liu Z.W., Haq I.U., Rabail R., Faheem F., Walayat N., Nawaz A., Shabbir M.A., Munekata P.E.S., Lorenzo J.M. (2022). Functional and nutraceutical significance of amla (*Phyllanthus emblica* L.): A review. Antioxidants.

[3] MoFPI (2021). Study on Identification of Gaps in Infrastructure and Processing Facilities to Develop Potential Value Chain of Amla. https://www.mofpi.gov.in/sites/default/files/study_on_infrastructure_gaps_-_amla.pdf.

[4] Ikram A., Khalid W., Aziz M., Arif M.A., Jha R.P., Khalid M.Z., Fizza C., Mehmood M.Z., Haseeb M., Rahim M.A. (2021). Nutritional and bio-chemical composition of amla (*Emblica officinalis*) and its therapeutic impact: A review. Acta Sci. Nutr. Health.

[5] Selvamuthukumaran M. (2020). Amla (*Indian Gooseberry*): Characteristics, Therapeutic Potential, and Its Value Addition. Asian Berries.

[6] Sawant S.S., Lee B., Song J., Seo H.J. (2022). The Indian gooseberry (*Emblica officinalis*) industry and cultivation in India. J. Korean Soc. Int. Agric..

[7] Gautam A.K., Bhadauria R. (2010). Fungal and mycotoxin contamination of some common stored herbal fruit samples. J. Indian Bot. Soc..

[8] Sengupta P., Sen S., Mukherjee K., Acharya K. (2020). Postharvest diseases of Indian gooseberry and their management: A review. Int. J. Fruit Sci..

[9] Martău G.A., Călinoiu L.F., Vodnar D.C. (2021). Bio-vanillin: Towards a sustainable industrial production. Trends Food Sci. Technol..

[10] Kurabetta L.K., Masti S.P., Gunaki M.N., Hunashyal A.A., Chougale R.B., Dalbanjan N.P., Kumar S.P. (2025). Vanillin reinforced cationic starch/poly (vinyl alcohol) based antimicrobial and antioxidant bioactive films: Sustainable food packaging materials. Sustain. Food Technol..

[11] de Oliveira Filho J.G., Miranda M., Ferreira M.D., Plotto A. (2021). Nanoemulsions as edible coatings: A potential strategy for fresh fruits and vegetables preservation. Foods.

[12] Taouzinet L., Djaoudene O., Fatmi S., Bouiche C., Amrane-Abider M., Bougherra H., Rezgui F., Madani K. (2023). Trends of nanoencapsulation strategy for natural compounds in the food industry. Processes.

[13] Thakur D., Rana P., Singh S.K., Bakshi M., Kumar S., Singh S. (2024). Nanoemulsion edible coating for shelf-life improvement and quality control in perishable products. Plant Nano Biol..

[14] Shiekh R.A., Malik M.A., Al-Thabaiti S.A., Shiekh M.A. (2013). Chitosan as a novel edible coating for fresh fruits. Food Sci. Technol. Res..

[15] Chen Q., Qi Y., Jiang Y., Quan W., Luo H., Wu K., Li S., Ouyang Q. (2022). Progress in research of chitosan chemical modification technologies and their applications. Mar. Drugs.

[16] Wu J., Zhang Y., Zhang F., Mi S., Yu W., Sang Y., Wang X. (2025). Preparation of chitosan/polyvinyl alcohol antibacterial indicator composite film loaded with AgNPs and purple sweet potato anthocyanins and its application in strawberry preservation. Food Chem..

[17] Yoksan R., Jirawutthiwongchai J., Arpo K. (2010). Encapsulation of ascorbyl palmitate in chitosan nanoparticles by oil-in-water emulsion and ionic gelation processes. Colloids Surf. B Biointerfaces.

[18] Hosseini S.F., Zandi M., Rezaei M., Farahmandghavi F. (2013). Two-step method for encapsulation of oregano essential oil in chitosan nanoparticles: Preparation, characterization and in vitro release study. Carbohydr. Polym..

[19] Chaudhary M.M., Patel D.S., Chaudhary D.H., Dighule S.B. (2020). Isolation and characterization of fungi associated with deterioration of papaya fruits. J. Pharmacogn. Phytochem..

[20] Geethanjali R., Aathiya V., Pratibha K.Y., Banu M. (2023). Antifungal assay by poisoned food technique. World J. Pharm. Res..

[21] Tomar M.S., Pradhan R.C. (2024). Effect of storage temperatures on physicochemical, textural, bioactive, and microstructure changes in amla fruit. J. Food Meas. Charact..

[22] Braich A.K., Kaur G., Singh A., Dar B.N. (2022). Amla essential oil-based nano-coatings of Amla fruit: Analysis of morphological, physiochemical, enzymatic parameters, and shelf-life extension. J. Food Process. Preserv..

[23] Parashar S. (2014). Comparison and biochemical estimation of three primary metabolites of medicinally important plant amla (*Phyllanthus emblica*). Int. J. Educ. Res. Rev..

[24] Padhi S., Dwivedi M. (2022). Physico-chemical, structural, functional and powder flow properties of unripe green banana flour after the application of Refractance window drying. Future Foods.

[25] Rai S., Singh S., Pathak N., Sharma S. (2024). Prolongation of the shelf life of amla fruit through the application of edible coatings and assessment of antibacterial and antioxidant attributes of coatings. Biochem. Cell. Arch..

[26] Detsi A., Kavetsou E., Kostopoulou I., Pitterou I., Pontillo A.R.N., Tzani A., Christodoulou P., Siliachli A., Zoumpoulakis P. (2020). Nanosystems for the encapsulation of natural products: The case of chitosan biopolymer as a matrix. Pharmaceutics.

[27] Maleki G., Woltering E.J., Mozafari M.R. (2022). Applications of chitosan-based carrier as an encapsulating agent in food industry. Trends Food Sci. Technol..

[28] Prasad J., Das S., Maurya A., Soni M., Yadav A., Singh B., Dwivedy A.K. (2023). Encapsulation of *Cymbopogon khasiana* × *Cymbopogon pendulus* essential oil (CKP-25) in chitosan Nanoemulsion as a green and novel strategy for mitigation of fungal association and aflatoxin B1 contamination in food system. Foods.

[29] El-Saadony M.T., Saad A.M., Sitohy M., Alkafaas S.S., Dladla M., Ghosh S., Mohammed D.M., Soliman T.N., Ibrahim E.H., Fahmy M.A. (2025). Chitosan nanoparticles: Green synthesis, biological activities, and sustainable frontiers in targeted drug delivery and cancer nanomedicine–A comprehensive review. Mater. Today Bio.

[30] Das S., Singh V.K., Dwivedy A.K., Chaudhari A.K., Deepika, Dubey N.K. (2021). Eugenol loaded chitosan nanoemulsion for food protection and inhibition of Aflatoxin B1 synthesizing genes based on molecular docking. Carbohydr. Polym..

[31] Soleymanfallah S., Khoshkhoo Z., Hosseini S.E., Azizi M.H. (2022). Preparation, physical properties, and evaluation of antioxidant capacity of aqueous grape extract loaded in chitosan-TPP nanoparticles. Food Sci. Nutr..

[32] Das S., Singh V.K., Chaudhari A.K., Dwivedy A.K., Dubey N.K. (2022). Efficacy of *Cinnamomum camphora* essential oil loaded chitosan nanoemulsion coating against fungal association, aflatoxin B1 contamination and storage quality deterioration of *Citrus aurantifolia* fruits. Int. J. Food Sci. Technol..

[33] Cai M., Wang Y., Wang R., Li M., Zhang W., Yu J., Hua R. (2022). Antibacterial and antibiofilm activities of chitosan nanoparticles loaded with *Ocimum basilicum* L. essential oil. Int. J. Biol. Macromol..

[34] Jiang X., Yu Y., Ma S., Li L., Yu M., Han M., Yuan Z., Zhang J. (2024). Chitosan nanoparticles loaded with *Eucommia ulmoides* seed essential oil: Preparation, characterization, antioxidant and antibacterial properties. Int. J. Biol. Macromol..

[35] Hasheminejad N., Khodaiyan F., Safari M. (2019). Improving the antifungal activity of clove essential oil encapsulated by chitosan nanoparticles. Food Chem..

[36] Froiio F., Ginot L., Paolino D., Lebaz N., Bentaher A., Fessi H., Elaissari A. (2019). Essential oils-loaded polymer particles: Preparation, characterization and antimicrobial property. Polymers.

[37] Hellali D.H., Ayachi N. (2023). Nanoencapsulation of Rosmarinus officinalis essential oil into Chitosan crosslinked to Tripolyphosphate nanoparticles and Alginate/Chitosan nanoparticles: Formulation, characterization, in vitro release study, and in vivo evaluation. J. Drug Deliv. Ther..

[38] Hadidi M., Pouramin S., Adinepour F., Haghani S., Jafari S.M. (2020). Chitosan nanoparticles loaded with clove essential oil: Characterization, antioxidant and antibacterial activities. Carbohydr. Polym..

[39] Danaei M., Dehghankhold M., Ataei S., Hasanzadeh Davarani F., Javanmard R., Dokhani A., Khorasani S., Mozafari Y.M. (2018). Impact of particle size and polydispersity index on the clinical applications of lipidic nanocarrier systems. Pharmaceutics.

[40] Yousefi M., Mohammadi V.G., Shadnoush M., Khorshidian N., Mortazavian A.M. (2022). *Zingiber officinale* essential oil-loaded chitosan-tripolyphosphate nanoparticles: Fabrication, characterization and in-vitro antioxidant and antibacterial activities. Food Sci. Technol. Int..

[41] Babar H., Wu H., Zhang W., Asim M., Koşar A. (2025). Synergistic approach to colloidal stability and thermophysical optimisation of multi-walled carbon nanotubes, aluminium nitride, and silver-based hybrid nanofluids. Powder Technol..

[42] Karmakar S. (2019). Particle size distribution and zeta potential based on dynamic light scattering: Techniques to characterize stability and surface charge distribution of charged colloids. Recent Trends Mater. Phys. Chem..

[43] Oliveira Lima K., Barreto Pinilla C.M., Alemán A., López-Caballero M.E., Gómez-Guillén M.C., Montero P., Prentice C. (2021). Characterization, bioactivity and application of chitosan-based nanoparticles in a food emulsion model. Polymers.

[44] Alcantara K.P., Pajimna R.M.B., Aliga P.J.S., Malabanan J.W.T., Tangwongsiri C., Haworth I.S., Rojsitthisak P. (2025). Review of Chitosan-Coated Nanoscale Liposomes for Enhanced Drug Delivery. ACS Appl. Nano Mater..

[45] Souza M.P., Vaz A.F., Correia M.T., Cerqueira M.A., Vicente A.A., Carneiro-da-Cunha M.G. (2014). Quercetin-loaded lecithin/chitosan nanoparticles for functional food applications. Food Bioprocess Technol..

[46] Azadi A., Rafieian F., Sami M., Rezaei A. (2023). Fabrication, characterization and antimicrobial activity of chitosan/tragacanth gum/polyvinyl alcohol composite films incorporated with cinnamon essential oil nanoemulsion. Int. J. Biol. Macromol..

[47] Ahmed H., Noyon A.R., Uddin E., Rafid M., Hosen S., Layek R.K. (2025). Development and characterization of chitosan-based antimicrobial films: A sustainable alternative to plastic packaging. Clean. Chem. Eng..

[48] Baghel S., Cathcart H., O’Reilly N.J. (2016). Polymeric amorphous solid dispersions: A review of amorphization, crystallization, stabilization, solid-state characterization, and aqueous solubilization of biopharmaceutical classification system class II drugs. J. Pharm. Sci..

[49] Budiman A., Ivana H., Huang K.A., Huang S.A., Nadhira M.S., Rusdin A., Aulifa D.L. (2025). Biocompatible natural polymer-based amorphous solid dispersion system improving drug physicochemical properties, stability, and efficacy. Polymers.

[50] Mirsharifi S.M., Sami M., Jazaeri M., Rezaei A. (2023). Production, characterization, and antimicrobial activity of almond gum/polyvinyl alcohol/chitosan composite films containing thyme essential oil nanoemulsion for extending the shelf-life of chicken breast fillets. Int. J. Biol. Macromol..

[51] Guerrero A.d.O.e.S., da Silva T.N., Cardoso S.A., da Silva F.F.F., de Carvalho Patricio B.F., Gonçalves R.P., Weissmuller G., El-Cheikh M.C., Carneiro K., Barradas T.N. (2024). Chitosan-based films filled with nanoencapsulated essential oil: Physical-chemical characterization and enhanced wound healing activity. Int. J. Biol. Macromol..

[52] Todorov J., Pantić M., Kozarski M., Lazić V., Todorović N., Obradović M., Daković A., Krajišnik D., Milašinović N., Mirković M. (2025). Chitosan–Glucan Biopolymer Design: Extraction from Champignons with Improved Antioxidant and Antimicrobial Features. Processes.

[53] Phuong N.T.H., Koga A., Nkede F.N., Tanaka F. (2023). Application of edible coatings composed of chitosan and tea seed oil for quality improvement of strawberries and visualization of internal structure changes using X-ray computed tomography. Prog. Org. Coat..

[54] Severino P., da Silva C.F., da Silva M.A., Santana M.H., Souto E.B. (2016). Chitosan cross-linked pentasodium tripolyphosphate micro/nanoparticles produced by ionotropic gelation. Sugar Tech.

[55] Amiri A., Mousakhani-Ganjeh A., Amiri Z., Guo Y.G., Singh A.P., Kenari R.E. (2020). Fabrication of cumin loaded-chitosan particles: Characterized by molecular, morphological, thermal, antioxidant and anticancer properties as well as its utilization in food system. Food Chem..

[56] Kayaci F., Uyar T. (2011). Solid inclusion complexes of vanillin with cyclodextrins: Their formation, characterization, and high-temperature stability. J. Agric. Food Chem..

[57] Jamil B., Abbasi R., Abbasi S., Imran M., Khan S.U., Ihsan A., Javed S., Bokhari H. (2016). Encapsulation of cardamom essential oil in chitosan nano-composites: In-vitro efficacy on antibiotic-resistant bacterial pathogens and cytotoxicity studies. Front. Microbiol..

[58] Yousefi M., Khanniri E., Sohrabvandi S., Khorshidian N., Mortazavian A.M. (2023). Encapsulation of *Heracleum persicum* essential oil in chitosan nanoparticles and its application in yogurt. Front. Nutr..

[59] Mohammed M.A., Syeda J.T., Wasan K.M., Wasan E.K. (2017). An overview of chitosan nanoparticles and its application in non-parenteral drug delivery. Pharmaceutics.

[60] Negi A., Kesari K.K. (2022). Chitosan nanoparticle encapsulation of antibacterial essential oils. Micromachines.

[61] Soni M., Paul K.K., Yadav M., Agnihotri P., Tiwari P., Dwivedy A.K. (2025). Enhancing the shelf-life of strawberry using chitosan-based edible coating of *Ocimum gratissimum* L. essential oil nanoemulsion. Plant Nano Biol..

[62] Akhter A., Shirazi J.H., Khan H.M.S., Hussain M.D., Kazi M. (2024). Development and evaluation of nanoemulsion gel loaded with bioactive extract of *Cucumis melo* var. *agrestis*: A novel approach for enhanced skin permeability and antifungal activity. Heliyon.

[63] Hasanin M.S., Ayoob F.A., Hashem A.H., Emam M. (2025). Synthesis of Chitosan based nanoemulsions and their characterization and antifungal activity toward fungi causing mucormycosis. Sci. Rep..

[64] KORE V.T., Devi H.L., Haque S., Kabir J. (2012). Post Harvest Treatments, Packaging, Storage and Value Addition of Aonla: An Overview. https://www.cabidigitallibrary.org/doi/pdf/10.5555/20133355701.

[65] Ahlawat P., Kumari A. (2025). Physicochemical Alterations in Fruits Caused by Edible Coatings during Storage: A Review. Agric. Rev..

[66] Showkat S., Anjum N., Ayaz Q., Mustafa S., Malik A.R., Beigh M.A., Banday N., Gulzar B., Wani S.M. (2025). Enhancing shelflife of plum fruit by chitosan-based nanoemulsion coating incorporated with ginger essential oil. Appl. Food Res..

[67] Das S.K., Vishakha K., Das S., Ganguli A. (2023). Antibacterial and antibiofilm activities of nanoemulsion coating prepared by using caraway oil and chitosan prolongs the shelf life and quality of bananas. Appl. Food Res..

[68] Al-Farsi M., Al-Hoqani H., Ali H.M., Al-Hattali D., Shah Y.A., Al-Abri R. (2025). Enhancing the Shelf-Life of Date Fruits by Application of Chitosan-Based Nanoemulsion Enriched with Grape Seed Oil. J. Food Qual. Hazards Control.

[69] Kaur K., Dhillon W.S. (2015). Influence of maturity and storage period on physical and biochemical characteristics of pear during post cold storage at ambient conditions. J. Food Sci. Technol..

[70] Sebastian S., Bala K.L., Humar A. (2018). Effect of essential oil coatings and storage conditions on shelf life of guava (*Psidium guajava*) and amla (*Emblica officinalis*). Allahabad Farmer.

[71] Abeysuriya H.I., Bulugahapitiya V.P., Jayatissa L.P. (2024). Variation of vitamin C content and antioxidant capacities during the post-harvest storage of fresh fruits under different temperatures. J. Stored Prod. Res..

[72] Yang R., Miao J., Shen Y., Cai N., Wan C., Zou L., Chen C., Chen J. (2021). Antifungal effect of cinnamaldehyde, eugenol and carvacrol nanoemulsion against *Penicillium digitatum* and application in postharvest preservation of citrus fruit. LWT.

[73] Yang J., Song J., Liu J., Dong X., Zhang H., Jeong B.R. (2024). Prolonged post-harvest preservation in lettuce (*Lactuca sativa* L.) by reducing water loss rate and chlorophyll degradation regulated through lighting direction-induced morphophysiological improvements. Plants.

[74] Petriccione M., Mastrobuoni F., Pasquariello M.S., Zampella L., Nobis E., Capriolo G., Scortichini M. (2015). Effect of chitosan coating on the postharvest quality and antioxidant enzyme system response of strawberry fruit during cold storage. Foods.

[75] Davis J., Clark S. (2024). The effect of chitosan edible coating on the respiration rate and ethylene production of guava. Int. J. Adv. Chem. Res..

[76] Kumari P. (2017). Evaluation of Chlorophyll and Cellulose Content in Different Varieties of Aonla during Room Temperature Storage. Chem. Sci. Rev. Lett..

[77] Dave R.K., Ramana Rao T.V., Nandane A.S. (2017). Improvement of post-harvest quality of pear fruit with optimized composite edible coating formulations. J. Food Sci. Technol..

[78] Moradinezhad F., Adiba A., Ranjbar A., Dorostkar M. (2025). Edible coatings to prolong the shelf life and improve the quality of subtropical fresh/fresh-cut fruits: A review. Horticulturae.

[79] Saleem M.S., Anjum M.A., Naz S., Ali S., Hussain S., Azam M., Sardar H., Khaliq G., Canan I., Ejaz S. (2021). Incorporation of ascorbic acid in chitosan-based edible coating improves postharvest quality and storability of strawberry fruits. Int. J. Biol. Macromol..

[80] Iqbal S.Z., Waseem M., Zia K.M., Iqbal M., Mohammed O.A., Amina N., Cui G., Doghish A.S., Abdel-Reheimand M.A., Khaneghah A.M. (2025). Application of chitosan, zinc oxide nanoparticles, and Aloe vera gel edible coating for the extension in shelf life of tomatoes. Food Packag. Shelf Life.

